# Supplementary material for: Dietary Cocoa Flavanols Enhance Mitochondrial Function in Skeletal Muscle and Modify Whole-Body Metabolism in Healthy Mice
Source: Nutrients. 2021 Sep 29;13(10):3466. doi: 10.3390/nu13103466 (PMC8538722; doi:10.3390/nu13103466)
Supplement: Supplementary file 1 [file nutrients-13-03466-s001.zip › nutrients-1383914-supplementary.pdf]

Supplemental data

Mitochondrial supercomplexes assessment:

Mitochondrial respiratory chain supercomplexes were assessed in isolated mitochondria from the white gastrocnemius muscle. Muscles were dissected from the surrounding connective tissue, rapidly removed, weighed, and placed in 10 mL of ice-cold mitochondrial isolation buffer (mM: 300 sucrose, 10 Tris HCl, 1 EGTA, and 5mg·mL^{-1} of BSA, pH 7.2). Muscles were minced with razor blades on a glace plate on ice for 2 min. Muscles were homogenised using a motor-driven Teflon pestle homogeniser, incubated 5 min with Nagarse protease (0.75g per g of tissue) and homogenised with the pestle homogeniser. The homogenate volume was completed to 40 mL and centrifuge at 800 x g for 10 min at 4 °C. The supernatant was decanted and centrifugated at 10, 000 x g for 10 min at 4 °C. The following step was realised two times: the pellet was resuspended in 40 mL of suspension buffer (mM: 300 sucrose, 10 Tris HCl, 0.05 EGTA, pH 7.2), and centrifuged at 7, 000 x g for 6 min at 4 °C. The final mitochondrial pellet was resuspended in 80 µL of suspension buffer, and protein concentrations were determined by the bicinchoninic acid method. The supercomplexes were identified as previously described [78]. Briefly, isolated mitochondria were exposed to the non-ionic detergent digitonin for 30 min, and after centrifugation, the supernatant was collected, quantified, and loaded in gradient gel. Electrophoretic separation was done at 150V for 1 h 30, the increased at 200V for 16 h in a cold room (4 °C). Gels were incubated in SDS solution for 1 h and transferred to PVDF membranes at 40 mA for 24 h. Membranes were blocked with Tris-buffered saline containing 1% (vol/vol) Tween-20 (TBS-T) and 5% (wt/vol) BSA and then probed overnight with primary antibodies (Complex I: 1/2000, #459100, Invitrogen; Complex II: 1/10 000, #Ab14715, Abcam; Complex III: 1/2000, #Ab14745, Abcam). Next, membranes were washed and probed with secondary antibodies (horseradish peroxidase-conjugated, 1/1000) for 2 h at room temperature. Membranes were then exposed to an enhanced chemiluminescent substrate for visualisation (ECL Kit; Thermo Scientific, Waltham, MA).

<u>Figure S1:</u> Immunoblot analysis of OXPHOS complexes following hybrid BN-PAGE. Replicates were electrotransferred on a single membrane. After transfer, individual lanes were cut and incubated with specific antibodies recognising CI, CII, and CIII. OXPHOS supercomplexes assemblies are identified using the standard nomenclature.

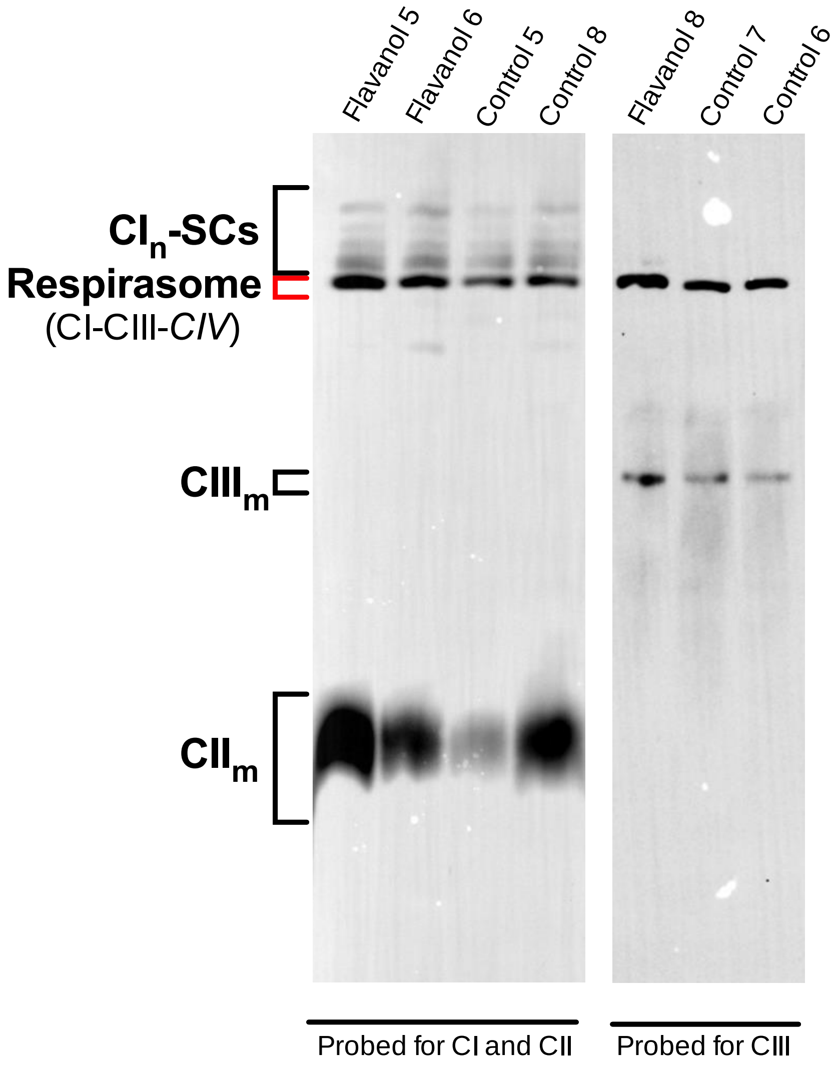

n = variable number
m = monomer
SCs = Supercomplexes
